# Evaluation of Frequency, Clinical Correlation, and Antibodies Confirmation Profile in Patients with Suspected Antiphospholipid Syndrome

**DOI:** 10.1055/s-0041-1736289

**Published:** 2021-10-19

**Authors:** Filipe F. Martins, Teresa M. L. Campos

**Affiliations:** 1 Department of Immunohemotherapy, Tâmega e Sousa Hospital Center, Penafiel, Portugal

**Keywords:** antiphospholipid syndrome, antiphospholipid antibodies, anticardiolipin, anti-β2-glycoprotein I, lupus anticoagulant, thrombosis

## Abstract

Antiphospholipid syndrome (APS) is a systemic autoimmune disease characterized by arterial and venous thrombotic manifestations and/or pregnancy-related complications in patients with persistent antiphospholipid (aPL) antibodies. The introduction of Sapporo's classification criteria allowed uniformity in the classification of this pathology, representing a considerable advance in its diagnosis. However, currently some doubts about the application of these criteria still persist. The aim of this study was to contribute to the better understanding of APS by the assessment of aPL prevalence, the association between clinical and laboratory tests, and evaluation of the aPL confirmatory profile.

In this study, 1,179 samples from patients with suspected APS of both genders, without age restrictions, who were advised to test for complete aPL's profile were analyzed. The samples were tested for lupus anticoagulant (LAC), anticardiolipin immunoglobulin (Ig) G/IgM and anti-β-2-glycoprotein I IgG/IgM antibodies. Patient samples with isolated test requests for analysis and samples from patients under the influence of anticoagulants or in an infectious process were excluded.

The overall positivity found was 17.9% and the most frequent aPL was LAC. The antibodies were determined in isolation and in association. The prevalence of triple positivity was 0.8% and double positivity was 1.8%. Positivity was higher in inpatient/emergency services compared with outpatient services. There was a higher positivity in individuals over 41 years, males, patients with systemic lupus erythematosus, kidney complications, and deep vein thrombosis/thrombophlebitis. The positivity confirmation with second sample was 39.5% and the confirmation profile shows that 50.6% of samples confirmed with same positivity profile; 17.3% with a different profile and regarding to these, 2.5% of the samples confirmed positivity with a different antibody from the previously detected.

This study suggests that the aPL's positivity tends to increase with age, showing that the aPL's testing should be avoided during an acute event and reinforces the need for complete aPL laboratory profile in the second sample and subsequent determinations.

## Introduction

Antiphospholipid syndrome (APS) is a systemic autoimmune disorder diagnosed based in clinical and laboratorial manifestations. 1 2 3 It is one of the most common acquired thrombophilia and it is associated with venous and arterial thrombosis. Thrombosis occurs most commonly in the deep veins of the leg and in cerebral arterial circulation 4 but might also occur less frequently in other locations such as the hepatic and visceral veins or cerebral venous circulation. 4 5 6 APS can also cause obstetric complications which include unexplained intrauterine deaths of a morphologically normal fetus caused by severe eclampsia or preeclampsia and/or spontaneous miscarriages. 6 A small number of individuals (<1%) may also develop catastrophic APS, characterized by multiple small-vessel occlusions that can lead to multiorgan failure. 7 8 Other clinical associations, which include thrombocytopenia, livedo reticularis, and skin ulcers, have also been associated with APS. 8 9

The diagnostic criteria currently used to classify this pathology are the Sapporo criteria, 10 reviewed in 2006 in Sydney. The updated classification criteria assume the presence of at least one clinical criteria (thrombosis or morbidity in pregnancy) and a laboratory positive result (lupus anticoagulant [LAC], anticardiolipin [aCL], or anti-β-2-glycoprotein I [aβ2GPI] antibodies). 6 Definitive classification criteria are established when at least one clinical and one laboratory positive result are present. 6 11 12 Laboratory tests should be confirmed at least 12 weeks after first determination to minimize the risk of making a diagnosis based on transient antiphospholipid (aPL) antibodies. 6

Although knowledge about the clinical manifestations of APS has evolved in recent years, the etiology remains unclear, making it difficult to predict who will develop APS, or what is the reason for its occurrence. Clinical manifestations are prevalent in the general population and may have a multifactorial etiology which makes laboratory investigation essential to the diagnosis of APS. 13 However, the correlation between laboratory tests and clinical manifestations of the disease is not completely clarified, 8 9 and even the laboratory tests used for APS detection and classification show several limitations regarding its robustness, reproducibility, standardization, and clinical relevance. 14 Since 2006, several studies approached the sensitivity and specificity of laboratory tests used in the diagnosis of APS and the correlation between the positivity levels and clinical disease manifestations have been made. However, some questions remain to be clarified. 15 16 17 Further studies approaching the laboratory aspect of APS and others that establish a correlation between clinical events and laboratory tests are required.

Therefore, this work aims to provide a contribution to the APS diagnosis problematic, from a laboratory perspective, assessing the aPL prevalence, determine the positivity profile by sample provenance, gender, age, and associated pathologies. The confirmation profiles in second sample will also be evaluated. Information from the association between clinical events and laboratory tests in the “Tâmega e Sousa” population can contribute to increase knowledge about the local reality of APS and help making clinical and laboratory decisions which can allow more rational and adequate use of the tests currently available.

## Methods

### Selection and Description of Participants

Data from patient samples with suspected APS analyzed in the Immunohemotherapy and Clinical Pathology laboratory services of Tâmega e Sousa Hospital Center, between February 2015 and January 2018, were collected. Patients of both genders, without age restrictions and with a request to complete the laboratory profile of APS that includes aCL immunoglobulin (Ig) G and IgM, aβ2GPI IgG and IgM, and LAC, were included in this study. Patient samples that requested for isolated tests for analysis and samples from patients under the influence of anticoagulants or in an infectious process were excluded.

### Technical Information

Blood samples collection and preparation: venous blood samples were collected by venipuncture in vacuum tubes. One 3.2% trisodium citrate tube and a serum-separating tube were collected from each patient (Greiner Bio-One GmbH, Frickenhausen, Germany). The citrate tubes were double-spin (first 15 minutes at 3,800 revolutions per minute [RPM], transferred to a secondary tube, centrifuged 10 minutes at 4,500 RPM) and processed by the ACL TOP autoanalyzer (Werfen Group, Barcelona, Spain). The samples collected in serum-separating tubes were centrifuged at 3,800 RPM for 10 minutes and processed by the Phadia 250 autoanalyzer (Thermo Fisher Scientific, Waltham, Massachusetts, United States).

Determination of aCL and aβ2GPI: analysis of aCL IgG and IgM and aβ2GPI IgG and IGM was performed by the Phadia 250 autoanalyser by the fluoroenzyme immunoassay method, using the commercial, validated ELIA Cardiolipin IgG and IgM and ELIA aβ2GPI IgG and IgM reagent kits (Thermo Fisher Scientific, Waltham, Massachusetts, United States). We used the Sydney criteria cut-off of >40 MPL-U/mL for aCL IgG and IgM. As there is no proposed Sydney criteria cut-off for aβ2GPI IgG and IgM, we used the cut-off values of >10 MPL-U/mL as suggested by the manufacturer. Results with low titers (doubtful) were not considered.

Determination of LAC: LAC was determined by the ACL TOP coagulometer using the commercial, validated reagent kits dilute Russell's viper venom time (DRVVT) screen and confirm silica clotting time (SCT) screen and confirm reagents (Werfen Group, Barcelona, Spain). The result was considered positive when the DRVVT and/or SCT normalized ratio was >1.2. Prothrombin time (PT) and activated partial thromboplastin time (aPTT) commercial, validated reagent kits were also used to discard other coagulation alterations not related to LAC. Recombiplastin 2G reagent was used to determine the PT and SynthASil reagent to measure aPTT (Werfen Group, Barcelona, Spain).

### Statistics

Statistical analysis was performed using IBM Statistical Package for the Social Sciences, version 21 for Windows. Statistical analysis included descriptive analysis, with absolute and relative frequencies, arithmetic means, and standard deviations. The results were presented in tables. When appropriate, Pearson's Chi-square test ( *χ* ^2^ ) was used to compare variables. A *ρ* value less than 0.05 were considered statistically significant.

## Results

This study included 1,179 samples from patients with suspected APS, age ranged between 9 and 88 years, and mean age was 46.8 (±14.9) years. Mean age was 45.2 (±14.8) and 50.5 (±14.6) years in females and males, respectively. Prevalence of females was higher with a female-to-male ratio of 2.4:1. The most frequent age group was between 41 and 60 years in both genders ( Table 1 ). From evaluated samples, 397 (33.7%) were from inpatient/emergency services and 782 (66.3%) from outpatient services.

**Table 1:** Sample distribution by gender and age

Age (y)	Females 70.6% ( *n* = 832)	Males 29.4% ( *n* = 347)	Total *n* = 1,179
≤20	1.5% ( *n* = 18)	0.7% ( *n* = 8)	2.2% ( *n* = 26)
21–40	26.3% ( *n* = 310)	5.8% ( *n* = 68)	32.1% ( *n* = 378)
41–60	32.3% ( *n* = 381)	16.1% ( *n* = 190)	48.4% ( *n* = 571)
61–80	9.4% ( *n* = 111)	5.8% ( *n* = 68)	15.2% ( *n* = 179)
>80	1.1% ( *n* = 12)	1.0% ( *n* = 13)	2.1% ( *n* = 25)

### Antiphospholipid Prevalence

To assess the overall antibodies prevalence, all patients with a complete laboratory profile of APS in first sample were selected. The sample prevalence from patients with at least one positive aPL was 17.9%. Evaluating the positivity for each aPL individually, it was found that LAC was the most frequently found (11.8%). Positivity for other aPL's was aβ2GPI IgM, 3.4%; aβ2GPI IgG, 3.0%; aCL IgM, 2.0%; and aCL IgG, 1.4%.

Assessing the positivity profile, it was found that antibodies can appear isolated or in association. The aPL's combinations found in the positive samples are shown in Table 2 . Evaluating the multiple positivity profiles in overall population, it was found that the percentage of samples with triple positivity was 0.8% and double positivity was 1.8%. Considering samples with double and triple positivity and including LAC, the percentage is 1.4%.

**Table 2:** Prevalence by positivity profile

Positivity profile *n* = 1,179		
**Triple positivity profile (LAC/aβ2GPI/aCL)**	% ( *n* )	
LAC/aβ2GPI IgG/aβ2GPI IgM/aCL IgG/aCL IgM	0.08 (1) a	0.8% (9)
LAC/aβ2GPI IgG/aβ2GPI IgM/aCL IgG	0.08 (1) a	
LAC/aβ2GPI IgG/aCL IgG	0.17 (2) a	
LAC/aβ2GPI IgM/aCL IgG/aCL IgM	0.08 (1) a	
LAC/aβ2GPI IgM/aCL IgG	0.26 (3) a	
LAC/aβ2GPI IgM/aCL IgM	0.08 (1) a	
**Double positivity profile**		
LAC/aβ2GPI IgG	0.42 (5) a	1.8% (21)
LAC/aβ2GPI IgM	0.26 (3) a	
aβ2GPI IgG/aβ2GPI IgM/aCL IgM	0.08 (1)	
aβ2GPI IgG/aCL IgG	0.34 (4)	
aβ2GPI IgG/aCL IgM	0.08 (1)	
aβ2GPI IgM/aCL IgM	0.59 (7)	
**Single positivity**		
LAC	10.43 (123)	15.3% (180)
aβ2GPI IgG	1.52 (18)	
aβ2GPI IgM	1.95 (23)	
aCL IgG	0.51 (6)	
aCL IgM	0.85 (10)	

Abbreviations: aβ2GPI, anti-β-2-glycoprotein I; aCL, anticardiolipin; Ig, immunoglobulin; LAC, lupus anticoagulant.

a Considering samples with double and triple positivity including LAC: 1.4%.

The aPL's prevalence in the first sample by sample source are shown in Table 3 . From 397 impatient/urgency samples, 21.4% were positive for at least one aPL, and in 782 samples from the outpatient, positivity was 16.0%. The significance between two groups allows us to conclude that there is a statistically significant difference in the overall positivity between inpatient/urgency and outpatient services ( *χ* ^2^ = 5,296; *ρ* = 0.021). Assessing the positivity prevalence to each aPL individually, it was found that the statistical difference was due to LAC ( *χ* ^2^ = 10.797; *ρ* =0.001). Positivity was higher in inpatient/urgency services (16.1%) when compared with outpatient services (9.6%). In the remaining aPL's, no statistically significant difference was found.

**Table 3:** aPL's positivity prevalence by sample source

aPL	Total ( *n* = 1,179) % ( *n* )	Impatient/urgency ( *n* = 397) % ( *n* )	Outpatient ( *n* = 782) % ( *n* )	*χ*^2^ ( *ρ* )
LAC	11.8 (139)	16.1 (64)	9.6 (75)	10.797 (0.001)
aβ2GPI IgM	3.4 (40)	3.0 (12)	3.6 (28)	0.250 (0.617)
aβ2GPI IgG	3.0 (35)	3.5 (14)	2.7 (21)	0.647 (0.421)
aCL IgM	2.0 (25)	1.8 (7)	2.3 (18)	0.368 (0.544)
aCL IgG	1.4 (16)	1.3 (5)	1.4 (11)	0.043 (0.836)
Overall a		21.4 (85)	16.0 (125)	5.296 (0.021)

Abbreviations: aβ2GPI, anti-β-2-glycoprotein I; aCL, anticardiolipin; aPL, antiphospholipid; Ig, immunoglobulin; LAC, lupus anticoagulant.

Note: A *ρ* value less than 0.05 was considered statistically significant.

a Considering samples with at least one positive aPL.

Regarding to aPL's positivity prevalence by age, we found a statistically significant difference ( *χ* ^2^ = 11.781; *ρ* = 0.019) with the highest prevalence of positive aPL's in the group aged over 41 years ( Table 4 ). Making the evaluation by gender, it was found that the aPL positivity was 23.6% in males and 15.4% in females. The male-to-female positivity ratio was 1.6:1. The significant evaluation in the two groups allows us to conclude that there is a statistically significant difference between gender and prevalence of aPL's positivity ( *χ* ^2^ = 11.375; *ρ* = 0.001). Evaluating the positivity prevalence to each aPL individually, only to LAC, statistically difference was found ( *χ* ^2^ = 10.166; *ρ* = 0.001) which was higher in males ( Table 5 ).

**Table 4:** Positivity prevalence by age

Age (y) % ( *n* )		Negative ( *n* = 969) % ( *n* )	With at least one positive antiphospholipid ( *n* = 210) % ( *n* )	*χ*^2^ ( *ρ* )
≤20	2.2 (26)	92.3 (24)	7.7 (2)	11.781 (0.019)
21–40	32.1 (378)	86.8 (328)	13.2 (50)	
41–60	48.4 (571)	78.8 (450)	21.2 (121) a	
61–80	15.2 (179)	82.1 (147)	17.9 (32) a	
>80	2.1 (25)	80.0 (20)	20.0 (5) a	

Note: A *ρ* value less than 0.05 was considered statistically significant.

a The observed frequency was higher than expected frequency.

**Table 5:** Positivity prevalence by gender

aPL	Males ( *n* = 347) % ( *n* )	Females ( *n* = 832) % ( *n* )	*χ*^2^ ( *ρ* )
LAC	16.4 (57)	9.9 (82)	10.166 (0.001)
aβ2GPI IgG	4.0 (14)	2.5 (21)	1.940 (0.164)
aβ2GPI IgM	4.3 (15)	3.0 (25)	1.298 (0.255)
aCL IgG	2.0 (7)	1.1 (9)	1.601 (0.206)
aCL IgM	2.3 (8)	2.0 (17)	1.081 (0.776)
Overall a	23.6 (82)	15.4 (128)	11.375 (0.001)

Abbreviations: aβ2GPI, anti-β-2-glycoprotein I; aCL, anticardiolipin; aPL, antiphospholipid; Ig, immunoglobulin; LAC, lupus anticoagulant.

Note: A *ρ* value less than 0.05 was considered statistically significant.

a Considering samples with at least one positive aPL.

To evaluate the aPL's prevalence by associated pathology, the samples were classified based on the clinical information provided by the patient's doctor. In individuals with multiple pathologies, the most relevant diagnosis for the study was considered. A wide variety of clinical manifestations were found ( Table 6 ). The highest prevalence of positive aPL's was found in SLE and kidney complications (40.0% in each), deep vein thrombosis (DVT)/thrombophlebitis (21.4%), and hematological manifestations (21.3%). Assessing the prevalence and clinical manifestations for each aPL individually, it was found that LAC was the most frequent antibody in all pathologies.

**Table 6:** Positivity by clinical manifestation and aPL's prevalence

Clinical manifestation		Stroke/TIA % ( *n* )	PTE % ( *n* )	DVT/thrombophlebitis % ( *n* )	Obstetric complications % ( *n* )	Inflammatory/osteoarticular disease % ( *n* )	Skin manifestations % ( *n* )	Hematological manifestations % ( *n* )	Ophthalmic complications % ( *n* )	Kidney complications % ( *n* )	SLE % ( *n* )	Other CSC % ( *n* )	Other CNS complications % ( *n* )	Other % ( *n* )
Prevalence by clinical manifestation ( *n* = 1,179)		25.5 (301)	6.1 (72)	8.7 (103)	11.0 (130)	14.1 (166)	7.7 (91)	4.0 (47)	2.1 (25)	0.8 (10)	1.3 (15)	3.1 (37)	6.2 (73)	8.7 (109)
With at least one positive aPL		18.9 (57)	13.9 (10)	21.4 (22)	14.6 (19)	18.7 (31)	12.1 (11)	21.3 (10)	20.0 (5)	40.0 (4)	40.0 (6)	27.0 (10)	24.7 (18)	6.4 (7)
aPL	LAC	13.6 (41)	6.9 (5)	16.5 (17)	10.0 (13)	12.0 (20)	9.9 (9)	10.6 (5)	12.0 (3)	30.0 (3)	26.7 (4)	16.2 (6)	13.7 (10)	2.8 (3)
	aβ2GPI IgG	3.3 (10)	2.8 (2)	3.9 (4)	3.1 (4)	2.4 (4)	1.1 (1)	2.1 (1)	0.0 (0)	10.0 (1)	6.7 (1)	0.0 (0)	8.2 (6)	0.9 (1)
	aβ2GPI IgM	2.3 (7)	2.8 (2)	3.9 (4)	0.0 (0)	4.8 (8)	2.2 (2)	6.4 (3)	0.0 (0)	10.0 (1)	6.7 (1)	13.5 (5)	5.5 (4)	2.8 (3)
	aCL IgG	0.7 (2)	0.0 (0)	1.9 (2)	2.7 (2)	2.4 (4)	0.0 (0)	4.3 (0)	8.0 (2)	0.0 (0)	0.0 (0)	0.0 (0)	2.7 (2)	0.9 (1)
	aCL IgM	3.0 (9)	4.2 (3)	1.9 (2)	0.8 (1)	3.0 (5)	0.0 (0)	0.0 (0)	0.0 (0)	0.0 (0)	6.7 (1)	2.7 (1)	1.4 (1)	1.8 (2)

Abbreviations: aβ2GPI, anti-β-2-glycoprotein I; aCL, anticardiolipin; aPL, antiphospholipid; CSC, circulatory system complications; CNS, central nervous system; DVT, deep vein thrombosis; Ig, immunoglobulin; LAC, lupus anticoagulant; PTE, pulmonary thromboembolism; SLE, systemic lupus erythematous; TIA, transient ischemic attack.

### Positivity Confirmation in Second Sample

To assess positivity in the second sample, only data from patients with at least one positive aPL determined in the first sample and that also evaluated in the second screen, at least 12 weeks apart from the first sample, were selected. It was considered that the positivity was confirmed whenever at least one aPL was detected in the second screen.

Among all patients who tested positive in the first sample ( *n* = 210), there were 132 (62.9%) patients who tested for the second time, while the positivity confirmation was not evaluated in 78 (37.1%). From 132 patients with second determination, 83 (39.5%) were positive and 49 (23.4%) were negative in the second testing. The positivity confirmation was also evaluated individually for each aPL ( Table 7 ) were which found that aPL's which presented greater confirmation frequency in second sample was aβ2GPI IgG (93.8%), aCL IgM (80.0%), and aβ2GPI IgM (73.1%).

**Table 7:** aPL determination in 132 aPL-positive patients at first determination (at time of event) with additional determination in the second sample

aPL	aPL first sample ( *n* = 132) % ( *n* )	aPL second sample ( *n* = 83) % ( *n* )
LAC	68.2 (90)	48.9 (44)
aβ2GPI IgM	19.7 (26)	73.1 (19)
aβ2GPI IgG	12.1 (16)	93.8 (15)
aCL IgM	11.4 (15)	80.0 (12)
aCL IgG	6.8 (9)	55.6 (5)

Abbreviations: aβ2GPI, anti-β-2-glycoprotein I; aCL, anticardiolipin; aPL, antiphospholipid; Ig, immunoglobulin; LAC, lupus anticoagulant.

In addition to isolated positivity analysis per aPL in second sample, the confirmation profile was also evaluated ( Table 8 ). For this, only data from patients with complete laboratory profile of APS in first and second samples were selected ( *n* = 81). Analyzing the results can be verified that, from the 81 samples evaluated, only 55 (67.9%) confirmed positive in second determination. Regarding the confirmation profile, it can be observed that 41 (50.6%) from 55 samples confirmed positive with the same profile. From the 14 (17.3%), who were confirmed positive by different profiles, the difference was caused by negative for one or more aPL's in 5 (6.2%), there was positive for new aPL's in 7 (8.6%), and the positivity was confirmed by a different aPL from previously detected in 2 (2.5%).

**Table 8:** Analysis of positivity confirmation profile in the second sample

Samples with positivity confirmation in second determination ( *n* = 81)		Confirmation profile compared with first sample			
Confirms positivity in second sample	67.9% (55)	Same profile	50.6% (41)		
		Different profile	17.3% (14)	Antibody negativation	6.2% (5)
				New antibody positivation	8.6% (7)
				Positivity by different antibody	2.5% (2)
Does not confirm positivity in second sample	32.1% (26)				

## Discussion

Our study was performed in a population suspected of APS of which the majority of individuals were not APS patients. No studies were found in the literature that report positive aPL's prevalence data in first determination and before the APS diagnostic. However, comparing our data with other studies that assessed aPL in patients with diagnosed APS, it appears that the prevalence of aPL's found in our population was lower for most antibodies. Les et al, in a study that includes 70 patients, observed that the LAC prevalence was 28.6%; aCL, 8.6%; and aβ2GPI, 4.3%. 18 In another study, Stojanovich et al, evaluating a population of 162 individuals with primary APS, reported that the prevalence of LAC was 53.7%; aβ2GPI IgG, 32.1%; aβ2GPI IgM, 44.4%; aCL IgG, 33.3%; and aCL IgM, 52.5%. 19 Fujieda et al, studying arterial thrombosis events in a Japanese population with APS, found a LAC prevalence of 82.3%; aβ2GPI IgG, 31.3%; aβ2GPI IgM, 7.1%; aCL IgG, 35.5%; and aCL IgM, 5.7%. 20 Although, the antibody prevalence in this study was generally lower compared with the literature, the distribution in positive aPL's is similar to those reported in previous studies. 9 In this study, and as found in others, LAC was the aPL most frequently found. In the literature, the frequency of other aPL's is quite variable, not allowing comparison.

In our population, the prevalence of samples with triple positivity was 0.8%, with double positivity was 1.8%, and with double and triple positivity and including LAC was 1.4%. Clinical studies found in literature confirm that presence of triple positivity in patients with APS, or in patients only with aPL's, represents an increased risk of thrombotic and/or obstetric events, especially when in association with LAC. 21 22 23 24 25 Yelnik et al, studying the persistence of triple positivity as a thrombotic risk factor in 98 patients, concluded that thrombotic risk was higher in patients with triple positivity. 26 Les et al also associated the persistence of multiple positivity with high thrombotic risk. 18 Pengo et al, evaluating the thrombotic risk associated with LAC, also concluded that thrombotic risk is lower in patients with only LAC positive, when compared others with triple positivity. 24 Patients with double positivity, but negative for LAC, have a lower risk. Probably, because in these patients, the level of aβ2GPI is not sufficient to induce the LAC positivity. 27 Patients with isolated positivity are less prone to develop events related with aPL's. However, in APS related with obstetric complications and arterial thrombosis, LAC positivity was considered the main predictor, even without other associated aPL's. 6

The highest positivity prevalence observed among inpatients compared with outpatients, indicates that aPL's may be associated with acute disease. These findings are in accordance with the literature recommendations which advise that, ideally, the aPL's determination should be done when patient is clinically stable and not during an acute event. The laboratory test results interpretation performed near an acute thromboembolic event can be difficult, since the acute phase factors, such factor VIII and fibrinogen, tend to be increased which may interfere with the coagulometric test results. In addition, acute events can induct the appearance of transient aCL's. 28 29 If aPL's determination is performed in the acute phase of the disease, the guidelines recommend confirmation of positive results to differentiate whether aPL's are persistent or transient. 28 30

The aPL prevalence was higher in males and in the group aged over 41 years, suggesting that the aPL's positivity tends to increase with age. However, in our population the mean age observed in males was higher than females that may have influenced the data age related in males. It is difficult to establish a comparison between aPL's prevalence by gender and age with other studies because the vast majority of studies reported the APS incidence data and not the aPL's prevalence found. Asherson et al reported a primary APS ratio between women and men of 2:1 31 and Font et al reported a ratio of 5:1. 32 Jara et al, in a study including 30 men and 38 women, reported a higher percentage of positive LAC in females. For aCL IgG and IgM, they found a higher positivity in the males. 33 With regard to APS incidence, Garcia et al, in a population study with adults, determined that the APS incidence was identical in both genders and more prevalent in the population over 75 years. 11 Manukyan et al, in a study performed in Germany and involving 5,000 individuals, found a strong association between the aCL and aβ2GPI IgM increased titers and age. They also found that the aPL's IgG titers tend to stabilize or decrease with the aging process. 34 Hwang et al reported that man showed aPL peak incidence rates at 70 to 79 years of age, while woman revealed peak incidence in 30 to 39 and 70 to 79 years of age. 35 The data performed by these authors seem to contradict our data for positivity by gender and corroborate with positivity related to age.

We found that LAC was the most frequent antibody in all pathologies. These results are in accordance with those of other studies found in literature. Wahl et al performed a meta-analysis with adult patients in 18 studies and demonstrated that, in SLE patients, the risk of thrombotic events is more strongly associated with LAC than other aPL's. 36 In another review, including 25 studies that encompassed several pathologies, Galli et al confirmed that LAC positivity represents an increased thrombotic risk factor. 37 Most recently, Male et al reported an association of LAC with thrombotic events, even stronger than that reported by Wahl et al. 38 Assessing the remaining aPL's positivity, it can be observed that the prevalence found was much lower than those found for LAC in all pathologies, and there seems to be no positivity pattern between aCL and aβ2GPI and different pathologies.

With regard to positivity confirmation in second sample, in this study, the aβ2GPI IgG was the aPL with higher percentage of confirmation, while LAC shows the lowest. In literature, the transient character of aPL's is documented 17 26 38 ; however, there are a few studies that provide data about aPL's behavior over time, and those that exist, reported follow-up data on patients with diagnosed APS. No studies were found in literature that determined the seroconversion frequency (between first and the second determinations) and before APS diagnostic. Medina et al, studying the thrombotic risk of hypocoagulation suspension in patients who became aPL's negative, determined that, in this population, 60 months after the initial diagnosis of APS, aPL's disappeared in approximately 50% of patients. 39 In another study, involving 105 women and a followed-up period of 10 years, Riancho-Zarrabeitia et al reported that 25% maintained a persistent positivity throughout the study and 50% became negative. They also found that, after becoming negative, these antibodies remained persistently negative. 40 Martinez-Berriotxoa and collaborators, in a cohort study involving 237 patients with SLE, demonstrated that positivity only remains persistent in 10% of patients. 17 Contrary to this, Erkan et al demonstrated that aPL's remain stable in at least 75% of patients for a 1 year to 3.5 years period depending on the test. 41 Most recently, Yelnik et al, assessing the thrombotic risk associated with triple positivity, demonstrated that after 13 years, 27% of patients with positive aPL's became negative, 10% positive for at least one more aPL and 9% for two aPL's. They also concluded that, aCL's were the antibodies that negativized most frequently and patients with triple positivity remain more stable over time. 26 The data reported by Yelnik et al corroborate with some of the results obtained in our study. It is widely accepted that the aPL's titers may vary over time and that variation can be caused by several factors. Transient aPL's can be inducted by infections as cytomegalovirus, 42 43 by pregnancy 16 44 and by inflammatory conditions in general and particular SLE. 45 On the other hand, some treatments, such as hydroxychloroquine and corticosteroid treatments, seem to decrease aPL's levels.

The data from our study allow us to conclude that confirmatory profile was quite variable, not allowing to identify a uniform trend of aPL's appearance or disappearance. However, our data reinforces the recommendations by Devreese et al that strongly emphasized that positive tests should be repeated at least 12 weeks after the initial positive test to exclude false positives, clinically unimportant, or transient antibodies. 30 Based in our data, we can add to this recommendation that all laboratory repetitions should include complete APS profile, that includes LAC, aCL IgG, aCL IgM, aβ2GPI IgG, and aβ2GPI IgM.

### Study Limitations

This study had some limitations inherent to its retrospective feature. One of the limitations was the loss of “follow-up” in second samples from patients with positive aPL's. In addition, it was not assessed if patients received corticosteroids treatment between first and second determinations. Clinical diagnostic also presented limitations. Diagnoses were made by the patient's doctor, so different diagnostic criteria may have been used in each pathology. Finally, there was no laboratory homogeneity in all samples, in the time that mediates clinical symptoms and laboratory determination. The same applies to laboratory determinations between the first and second samples.

## Conclusion

The intent of this study was to approach APS syndrome from a laboratorial perspective, trying correlate the aPL data with clinical events. Of the most relevant data obtained, we highlight the followings:

- The higher prevalence of aPL's observed in hospitalized patients shows that aPL's can be associated with acute disease states which reinforces the recommendation that, ideally, the determination of aPL's should be made when patient is clinically stable and not during an acute event. If the determination of aPL's is made during the acute disease, the positive results must be repeated at least 12 weeks after the initial determination.
- Regarding the positivity confirmation, and according to our research, our study was the first one that evaluates the seroconversion and the antibody positivity confirmation profile between first and subsequent determinations and before APS diagnostic. Based on our data, it can be added to our previous recommendation that in second sample and in the subsequent determinations, complete laboratory APS profile must always be repeated and not only the positive aPL's detected in the first sample.
- The highest prevalence of positive aPL's was found in the group aged over 41 years suggesting the aPL's positivity tends to increase with age.
- The LAC was the most commonly detected aPL in all clinical manifestations.

New studies that include a larger number of patients, with assessment of the complete laboratory APS profile in all determinations, may be useful to consolidate the data obtained in our study. The follow-up of patients, with a regular aPL's laboratory evaluation, could also be useful to understand the behavior of aPL's over time. An aPL's screening in the general population could also be useful to understand the degree of thrombotic risk in the healthy population which has never suffered from any thrombotic and/or obstetric event.
